# The Vestibulo‐Ocular Reflex is Associated With Visuospatial Dysfunction in Patients With Parkinson's Disease

**DOI:** 10.1002/brb3.70453

**Published:** 2025-04-01

**Authors:** Yukang Kim, Tonghoon Woo, Seoui Kwag, Hyunsoh Park, Hanseob Kim, Kyoungwon Baik, Sun‐Uk Lee, Euyhyun Park, Chan‐Nyoung Lee, Gerard J. Kim, Ji‐Soo Kim

**Affiliations:** ^1^ Neurotology and Neuro‐ophthalmology Laboratory Korea University Medical Center Seoul South Korea; ^2^ Department of Computer Science and Engineering Korea University Seoul South Korea; ^3^ Department of Neurology Korea University Medical Center Seoul South Korea; ^4^ Department of Neurology Seoul National University College of Medicine Seoul South Korea; ^5^ Dizziness Center Clinical Neuroscience Center Department of Neurology Seoul National University Bundang Hospital Seongnam South Korea

**Keywords:** cognition, head‐impulse test, Parkinson's disease, visuospatial function, vertigo

## Abstract

**Background:**

Visuospatial impairment is one of the most frequent cognitive deficits in patients with Parkinson's disease (PD). It remains unknown whether the vestibulo‐ocular reflex (VOR) function affects visuospatial perception and memory in patients with PD.

**Objective:**

To delineate the relationship between VOR and visuospatial function in patients with PD.

**Methods:**

We prospectively evaluated video head‐impulse tests in 151 patients with PD (mean age ± standard deviation, 68 ± 9 years; 77 male). All patients conducted the Rey Complex Figure test (RCFT).

**Results:**

RCFT‐copying and RCFT‐delayed recall were impaired in 11 (11/151, 7%) and 15 (15/151, 10%) patients, respectively. The VOR gain was normal in 55 patients with PD (55/151, 36%). However, 69 patients overestimated VOR gain for at least one canal, and 34 patients showed a decreased gain for at least one canal (seven patients showed an overestimated gain for some canals and decreased gain for other canals). Multivariable logistic regression analysis showed that abnormal RCFT‐copying was negatively associated with the VOR gain for the horizontal canal (odds ratio [OR]: 0.001, 95% confidence interval [CI]: 0.001–0.08, *p* = 0.007). In contrast, abnormal RCFT‐delayed recall was negatively associated with Mini‐Mental State Examination scores (0.70, 0.52–0.93, *p* = 0.013), positively with age (1.11, 1.00–1.22, *p* = 0.041), male sex (12.82, 1.17–142.86, *p* = 0.036), years of schooling (1.41, 1.09–1.82, *p* = 0.009), but not with the VOR gain for any canal.

**Conclusions:**

The VOR function may be associated with deficits in visuospatial perception and learning in patients with PD. This implicates the development of more targeted therapeutic interventions and offers insights into the broader implications of PD on sensory‐motor integration and cognitive function.

## Graphical abstract

Our findings suggest the vestibulo‐ocular reflex function may be associated with visuospatial perception and learning in Parkinson's disease.

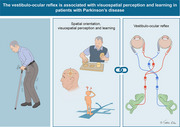

## 1 Introduction

A growing body of literature suggests that the vestibular system plays an important role in diverse cognitive processes, including visuospatial perception, memory, attention, and executive function (Bigelow et al. 2015; Oh et al. 2024). In particular, the vestibular system transduces angular and linear head accelerations, controls eye movements, posture, and balance, and participates in spatial perception (Cronin et al. 2017). Indeed, severance of the vestibular nerve impairs the visuospatial ability in animals (Smith and Zheng 2013). Similarly, patients with vestibular disorders may present with disabilities in spatial orientation and memory (Brandt et al. 2014). This trend was also replicated in healthy participants, showing an inverse correlation between vestibular function and spatial cognition performance (Bigelow et al. 2015).

Visuospatial impairment is among the most frequent cognitive deficits in PD and is observed in nearly all patients from the earliest stage (Aarsland et al. 2017; Hovestadt et al. 1987; Williams‐Gray et al. 2007). Visual dysfunction can be associated with navigational errors in PD regardless of the severity of motor symptoms, thereby affecting patients’ daily activity and quality of life (Uc et al. 2007). The origin of visuospatial dysfunction can be explained in many ways. It can result from inappropriate cues associated with executive dysfunction (Crucian et al. 2000). Patients with PD show poor performance on visuospatial tasks and fail to utilize cues to estimate verticality in space properly. This phenomenon was found regardless of the position of the head, suggesting the disruption of frontal‐basal ganglia being the culprit of visuospatial dysfunction (Crucian et al. 2000).

Alternatively, vestibular dysfunction may affect visuospatial ability in PD. The role of vestibular dysfunction has been explained by a bottom‐up theory that infers the basal ganglia may disrupt the vestibulo‐thalamo‐cortical connection, consequently affecting the visuospatial ability (White et al. 1983). It is supported by the fact that the perception of head verticality is associated with errors in line orientation in PD (Proctor et al. 1964). Contrastingly, the role of vestibular dysfunction is debated since visual perception bias is not dependent on vestibular modulation (Bronstein et al. 1996). In PD, the link between vestibular dysfunction and visuospatial disorientation is still unclear from this standpoint. Additionally, objective vestibular testing was not conducted to validate the association, as prior studies were performed before the introduction of video head‐impulse tests (video‐HITs). Thus, we investigated the results of video‐HITs to determine whether the VOR function is associated with visuospatial ability in patients with PD.

## 2 Methods

We prospectively recruited 181 consecutive patients with newly diagnosed PD between February 2022 and May 2024 at Korea University Medical Center. Video‐HITs were included in every patient's initial assessment.

The eligibility of the study was patients with newly diagnosed de‐novo PD, before the administration of PD‐specific medications, who underwent the rey complex figure test (RCFT) and video‐HITs properly as the initial assessment. Exclusion criteria include unavailable video‐HIT or RCFT due to poor compliance or artifacts. Patients with a prior diagnosis of peripheral or central vestibulopathy that can affect the results of video‐HITs were also excluded from the analysis.

According to the exclusion criteria, we excluded five patients whose video‐HITs were unavailable because of artifacts during impulses (large head bounce, exaggerated blinking, and lid and eye position artifacts). Those who failed to achieve optimal peak head acceleration due to excessive neck rigidity were also excluded (n = 8). Patients who did not undergo RCFT were excluded (n = 4). We further excluded patients with a history of central or vestibular disorders, including vestibular neuritis (n = 5), posterior circulation stroke (n = 4), Meniere's disease (n = 2), and vestibular migraine (n = 2). Finally, 151 patients (mean age ± SD = 68 ± 9 years; 77 male) were included for analysis.

### 2.1 Video‐HITs

Head and eye movements were recorded using video‐HITs (SLVNG, SLMED, Seoul, South Korea), as described previously (S.‐H. Kim et al. 2023). All patients discontinued any medication that may have affected the results of the video‐HITs at least 48 h before the test.

The patients were seated 1.2 m in front of the target. Eye position was calibrated using a red dot sequentially presented from the center at 10 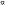 in the vertical and horizontal directions. Passive unpredictable, high‐acceleration, small‐amplitude head rotations were then delivered in the plane of the horizontal canals (HCs). After evaluating the HCs, head impulses for the vertical canals were delivered with the patient's head turned 30–40 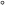 to the left or right of the fixation point eccentrically and aligned to the plane of the right anterior‐left posterior and left anterior‐right posterior canals. Eye and head movement data were synchronously sampled at a rate of 120 Hz. Eye movements were included in the analyses only when the peak head acceleration during HIT was > 2,500°/s^2^ for the HCs and 1,500°/s^2^ for the vertical canals (Woo et al. 2024). After excluding trials with blinks and outliers, at least 10 valid head impulses were recorded in each direction (Kim et al. 2023).

To measure the VOR gain, the ratio of the area under the eye velocity curve was compared to the area under the head velocity curve. The VOR gains for each canal were measured for individual impulse trials as the ratio of the mean velocity of the eye divided by that of the head during the 40‐ms time window centered at peak head acceleration (Kim et al. 2023). For statistical analyses, we calculated the mean gains for the HC, anterior canal (AC), and posterior canal (PC) on both sides.

The results of video‐HITs were determined to be positive (abnormal) when they fell outside the normal range of VOR gain from 18 healthy age‐, sex‐matched participants (mean age ± SD = 63 ± 7 years, 10 male) (Hong et al. 2024). The reference range was defined as mean ± 2 SD. The normal range of the VOR gain for the HC, AC, and PC were 0.86–1.20, 0.74–1.23, and 0.72–1.29, respectively. The eyelids were taped and lifted during the vertical impulses to prevent eyelid flicks as required (Yoon et al. 2024).

In addition to the VOR gain measurements, the presence of reversed catch‐up saccades was determined as previously described (J.‐G. Kim et al. 2022). The reversed catch‐up saccades were analyzed based on the velocity‐time graphs of the impulses in all canal planes. They were determined to be present when 1) they were directed toward the direction of head rotation and 2) the peak eye velocity of the corrective saccades exceeded 60°/s with a cutoff value according to the main sequence of the saccades (Leigh and Zee 2015).

### 2.2 Assessment of Visuospatial Function and Memory

Each patient underwent the RCFT‐copying test as a subdomain of the Seoul neuropsychological screening battery (Kang et al. 2003; Ryu and Yang 2023). Each patient was instructed to draw the RCFT again 20 min later, after frontal and executive function evaluation, to assess visuospatial memory (RCFT‐delayed recall). Both raw data and z‐scores were provided for the tests to judge their accuracy with respect to standardized values, as measured in the same age‐education group. RCFT‐copying and RCFT‐delayed recall were determined to be abnormal when the z‐scores were lower than 2 SD from the mean (*z‐*score < − 2.0). Mini‐mental state examination (MMSE) was conducted on all patients. Patients were also queried regarding psychotic and compulsive complications using the scale for outcomes in Parkinson's disease‐psychiatric complications (SCOPA–PC), with higher scores indicating more psychiatric complications.

### 2.3 Motion Analysis

Temporal and spatial gait characteristics were measured using a gait analyzer (GaitRite. 12 fit AP1105; CIR systems, Franklin, NJ, USA). Ten gait cycles were measured, and the average values were calculated. The detailed method has been previously described (Pyo et al. 2017). Among the various parameters, we analyzed cadence (steps/min), walking velocity (cm/s), and step length difference (cm).

### 2.4 Assessment of Parkinsonism

The severity of motor disability in patients with PD was assessed using the MDS–UPDRS motor part and the Hoehn and Yahr scale (Goetz et al. 2008).

### 2.5 Statistical Analysis

Nominal and independent variables were compared using the χ ^2^ or Fisher's exact test. Continuous and independent variables were compared using the Mann–Whitney U test and Spearman's correlation. For logistic regression analysis, significant variables were selected using the backward variable selection method. Statistical significance was set at *p* < 0.05 in the multivariable logistic regression analyses.

Statistical analyses were performed using the R software package (version 3.4.0; http://www.r‐project.org), and the statistical significance was set at a two‐tailed *p* < 0.05.

## 3 Results

### 3.1 Clinical Characteristics

Detailed clinical characteristics of the 151 patients are presented in Table 1. The disease duration ranged from 1 month to 12 years (median, 12 months; interquartile range [IQR], 6–18 months). The median MDS‐UPDRS‐III scores and H&Y scales were 23 (IQR = 16–31) and 2 (2–2.5), respectively. Patients had comorbidities, including dyslipidemia (n = 61), hypertension (n = 60), diabetes mellitus (n = 34), depressive disorder (n = 32), anxiety disorder (n = 19), history of coronary artery occlusive disease (n = 10), and transient ischemic attack or cerebrovascular disease (n = 6). The neurological sequelae of prior stroke were mostly minimal, with a modified Rankin scale score of 0 in three and 1 in two having dysarthria (n = 2) and hemiparesis (n = 2). None of the patients developed any red flag signs suggesting atypical Parkinsonian syndromes at the initial evaluation or during the follow‐up.

**TABLE 1:** Clinical characteristics of the patients.

Variables	Values
Age, mean ± SD, years	68 ± 9
Sex, female (%)	74 (49)
Body weight, mean ± SD, kg	63 ± 10
Disease duration, median (IQR), months^a^	12 (6–18)
MDS‐UPDRS‐III, median (IQR)	23 (16–31)
H&Y scale (%)	
1.0	21 (14)
1.5	5 (3)
2	72 (48)
2.5	32 (21)
3	19 (13)
4	2 (1)
VOR gain, median (IQR)	
RHC	1.07 (0.98–1.14)
LHC	1.01 (0.93–1.09)
RAC	1.08 (0.97–1.23)
LAC	1.09 (0.97–1.22)
RPC	1.04 (0.93–1.13)
LPC	1.03(0.93–1.15)
RCFT‐copying *t*‐score, median (IQR)	33 (29–35)
RCFT‐delayed recall *t*‐score, median (IQR)	12 (6–18)
SCOPA‐PC	3 (3–5)
Orthostatic hypotension (%)	54 (36)
RBD (%)	56 (37)
Restless leg syndrome (%)	13 (9)

^a^ Disease duration refers to the period from onset of motor symptoms to presentation.

Abbreviations: H&Y = Hoehn and Yahr, IQR = interquartile range, MDS‐UPDRS‐III = Movement Disorder Society‐Unified Parkinson's Disease Rating Scale motor part, RBD = Rapid eye movement sleep behavior disorder, RCFT = Rey Complex Figure test, SCOPA‐PC = Scale for Outcomes in Parkinson's disease‐Psychiatric Complication, SD = standard deviation, VOR = vestibulo‐ocular reflex.

### 3.2 Video‐HITs

The VOR gain was normal in 55 patients with PD (55/151, 36%; Tables 1 and 2). However, there was a decrease in the VOR gain at least for one semicircular canal in 34 patients (34/151): HC in 21 patients (right only in 8; left only in 7; both right and left in 6); any AC in 11 patients (right only in 6; left only in 1; both right and left in 4); and any PC in 16 patients (right only in 3; left only in 8; both right and left in 5). In contrast, 69 patients showed overestimation of the VOR gain for at least one canal plane (69/151, 46%; seven patients showed VOR overestimation in certain canal planes and decreased gain in other canals): any HC in 23 patients (right only, n = 10; left only, n = 5; both left and right, n = 8); any AC in 48 patients (right only, n = 16; left only, n = 12; both right and left, n = 20); and any PC in 13 patients (right only, n = 4; left only, n = 4; both right and left, n = 5). None of the patients exhibited reversed catch‐up saccades during the impulses. The VOR gain did not differ for any canal between the patients with abnormal RCFT‐copying and those normal (*p* = 0.550 for HC; *p* = 0.334 for AC; *p* = 0.985 for PC; Table 2).

**TABLE 2:** Clinical characteristics of patients with abnormal RCFT‐copying and those normal RCFT‐copying.

	Patients with abnormal RCFT‐copying (n = 11)	Patients normal RCFT‐copying (n = 140)	*p* value
Age, mean ± SD, years	71 ± 5	67 ± 9	*0.035*
Sex, female (%)	2 (18)	72 (51)	0.056
Body weight, mean ± SD, kg	65 ± 8	63 ± 10	0.399
Disease duration, median (IQR), months^a^	12 (6–30)	12 (6–18)	0.533
MDS‐UPDRS‐III, median (IQR)	31 (23–34)	22 (16–30)	0.066
H&Y scale, median (IQR)	2.5 (2–2.5)	2 (2–2.5)	*0.015*
MDS‐UPDRS‐III‐hand rigidity^a^	1 (1–2)	1 (1–2)	0.586
MDS‐UPDRS‐III‐finger tapping^a^	1 (1–1)	1 (1–2)	0.383
MDS‐UPDRS‐III‐kinetic tremor^a^	1 (1–1)	1 (0–1)	0.189
**Video‐HITs, median (IQR)**			
VOR gain, HC	0.98 (0.89–1.08)	1.05 (0.97–1.10)	0.191
VOR gain, AC	1.03 (0.92–1.15)	1.10 (0.99–1.19)	0.296
VOR gain, PC	1.07 (0.99–1.11)	1.04 (0.94–1.12)	0.794
**Motion analyses, mean** ± **SD**			
Walking velocity, cm/s	89.0 ± 17.8	90.5 ± 23.8	0.858
Walking cadence, step/min	113.8 ± 15.3	106.6 ± 11.7	0.097
Step length difference, cm	2.7 ± 2.1	2.3 ± 2.2	0.668
**Visuospatial and cognition**			
MMSE, median (IQR)	26 (21–27)	28 (27–29)	0.001
Years of schooling, median (IQR)	9 (6–12)	12 (6–16)	0.279
RCFT‐copying t‐score	23 (20–25)	33 (30–35)	< 0.001
RCFT‐copying z‐score	—2.7 ± 0.5	0 ± 0.8	< 0.001
RCFT‐delayed recall t‐score	4 (2–7)	13 (7–19)	0.001
RCFT‐delayed recall z‐score	—1.5 ± 0.8	—0.3 ± 1.1	0.001
SCOPA‐PC, median (IQR)	3 (3–5)	3 (3–5)	0.962
**Comorbidities**			
Orthostatic hypotension (%)	4 (36)	50 (36)	> 0.999
REM sleep behavior disorder (%)	5 (46)	51 (36)	0.551
Restless leg syndrome (%)	2 (18)	11 (8)	0.241
Diabetes mellitus (%)	3 (27)	31 (22)	0.687
Hypertension (%)	5 (46)	55 (39)	0.738
Dyslipidemia (%)	5 (46)	56 (40)	0.723
Cerebrovascular attack (%)	1 (9)	5 (4)	0.370
Coronary artery occlusive disease (%)	0 (0)	10 (7)	> 0.999
Depression (%)	1 (9)	31 (22)	0.459
Anxiety (%)	1 (9)	18 (13)	> 0.999
Sedative or vestibular suppressant drugs (%)	1 (9)	36 (26)	0.296

^a^ in dominant hand.

Abbreviations: CI = confidence interval, H&Y = Hoehn and Yahr, IQR = interquartile range, MDS‐UPDRS‐III = Movement Disorder Society‐Unified Parkinson's Disease Rating Scale motor part, MMSE = mini‐mental state examination, REM = Rapid eye movement, SCOPA‐PC = Scale for Outcomes in Parkinson's disease‐Psychiatric Complication, SD = standard deviation.

^a^Disease duration refers to the period from onset of motor symptoms to presentation.

### 3.3 Visuospatial Function and Cognition

The RCFT‐copying was impaired in 11 (11/151, 7%) and the RCFT‐delayed recall in 15 (15/151, 10%) patients (Table 2). Patients having abnormal RCFT‐copying had lower MMSE scores (*p =* 0.001), yet higher H&Y scale (*p* = 0.015) than those showing normal RCFT‐copying (Table 2).

The MDS–UPDRS–III score did not differ between the patients with normal and abnormal RCFT‐copying (*p* = 0.066). However, Patients with abnormal RCFT‐copying showed higher H&Y scores than those with normal RCFT‐copying (*p* = 0.015). The MDS‐UPDRS subscales of kinetic hand rigidity (*p* = 0.586), finger tapping (*p* = 0.383), and kinetic tremor (*p* = 0.189) did not differ between the two.

### 3.4 Correlation Between Video‐HITs and Visuospatial Function and Cognition

The RCFT‐copying z‐score had a weak positive correlation with the VOR gain for HC (*r* = 0.160, *p* = 0.049). However, no correlation was found for other vertical canals (*r* = 0.001, *p* = 0.992 for AC; *r* = ‐0.035, *p* = 0.667 for PC). The RCFT‐delayed recall *z*‐scores were not associated with the VOR gain for any canal (*r* = 0.079, *p* = 0.337 for HC; *r* = ‐0.027, *p* = 0.738 for AC; *r* = 0.005, *p* = 0.948 for PC).

The MMSE score was not correlated with the VOR gain for any canal (*r* = 0.100, *p* = 0.222 for HC; *r* = ‐0.069, *p* = 0.397 for AC; *r* = 0.090, *p* = 0.269 for PC).

### 3.5 Prediction of Visuospatial Dysfunction

Multivariable logistic regression analysis showed that abnormal RCFT‐copying was negatively associated with the VOR gain for the HC (odds ratio [OR] = 0.001, 95% confidence interval [CI] = 0.001–0.08, *p =* 0.007; Table 3).

**TABLE 3:** Prediction of abnormal RCFT‐copying using multiple logistic regression analyses.

Variables	Univariate analysis		Multivariable analysis	
	OR (95% CI)	*p*‐value	OR (95% CI)	*p*‐value
Age, years	1.05 (0.98–1.13)	0.195		
Sex, female	0.21 (0.04–1.01)	0.051	0.12 (0.01–1.32)	0.083
Disease duration, months^a^	1.01(0.99–1.04)	0.363		
MDS‐UPDRS‐III	1.04 (0.99–1.08)	0.150		
MDS‐UPDRS‐III‐kinetic tremor^b^	1.51 (0.71–3.22)	0.280		
Orthostatic hypotension	0.97 (0.27–3.48)	0.965		
Years of schooling, years	0.93 (0.81–1.07)	0.293		
MMSE	0.75 (0.62–0.89)	0.001		
SCOPA‐PC	1.18 (0.83–1.66)	0.360		
Depression	0.35 (0.04–2.85)	0.328		
Anxiety	0.68 (0.08–5.62)	0.718		
Cadence, steps/min	1.05 (0.99–1.12)	0.098		
VOR gain, HC	0.04 (0.001–3.59)	0.157	0.001 (0.001–0.08)	0.007
VOR gain, AC	0.25 (0.01–8.37)	0.448		
VOR gain, PC	0.91 (0.03–31.93)	0.960		

^a^ Disease duration refers to the period from onset of motor symptoms to presentation.

^b^ in dominant hand.

Abbreviations: AC = anterior canal, CI = confidence interval, HC = horizontal canal, H&Y = Hoehn and Yahr, MDS‐UPDRS‐III = Movement Disorder Society‐Unified Parkinson's Disease Rating Scale motor part, MMSE = mini‐mental state examination, OR = odds ratio, PC = posterior canal, RCFT‐copying = Rey Complex Figure test copying task, SCOPA‐PC = Scale for Outcomes in Parkinson's Disease‐Psychiatric Complications, VOR = vestibulo‐ocular reflex.

Abnormal RCFT‐delayed recall was inversely associated with MMSE scores (0.70, 0.52–0.93, *p* = 0.013) and positively with age (1.11, 1.00–1.22, *p* = 0.041), male sex (12.82, 1.17–142.86, *p* = 0.036), and years of schooling (1.41, 1.09–1.82, *p* = 0.009). In contrast, abnormal RCFT‐delayed recall was not associated with the VOR gain for the HC (*p* = 0.919), AC (*p* = 0.362), or PC (*p* = 0.533).

### 3.6 Sensitivity Analysis

(1) RCFT‐copy defined as z‐score < − 1.0

Due to an imbalanced distribution of outcomes regarding abnormality on RCFT (11 abnormal RCFT‐copying versus 140 normal), we conducted a sensitivity analysis setting a 1–SD threshold to define abnormality. Under this criterion, 32 patients were determined as being abnormal (32/151, 21%). The trend was similarly observed in multivariable logistic analysis, which indicated a negative association with disease duration, years of schooling, and VOR gain for the HC (Supplementary Table 1).

(2) Decreased VOR gain as nominal variable

As VOR overestimation in PD patients could bias the result, we set decreased VOR gain as nominal variables. The trend was similarly observed in multivariable logistic analysis, showing that decreased VOR gain for the HC is associated with abnormal RCFT‐copy (Supplementary Table 2).

## 4 Discussion

The findings of this study can be summarized as follows: (1) 7–21% of our patients showed abnormal results on RCFT‐copying, indicating visuospatial learning and perception dysfunction; (2) Abnormal RCFT‐copying test results were negatively associated with VOR gain for the HC; (3) Abnormal RCFT‐delayed recall was negatively associated with MMSE scores and positively with age, male sex, and years of schooling. No association was found with the VOR gain for any canal.

### 4.1 Cognition and Visuospatial Function in Patients With PD

Apart from visuospatial dysfunction, patients with PD can also show various cognitive deficits across attention, execution, and language (Ekker et al. 2017; Shin et al. 2022; Williams‐Gray et al. 2007). The spectrum of cognitive impairment ranges from subjective cognitive decline to dementia (Williams‐Gray et al. 2009; Williams‐Gray et al. 2013). Cognitive decline may not be specific to a certain domain but rather global as the disease progresses (Aarsland et al. 2017). Among various cognitive functions, visuospatial function can be impaired in the earliest stage (Aarsland et al. 2017; Hovestadt et al. 1987; Mosimann et al. 2004; Williams‐Gray et al. 2007). This deficit can be identified through cognitive tests of line orientation, spatial location memory, and mental rotation (Pereira et al. 2009). Furthermore, object detection, categorization of visual stimuli, and face recognition can be affected. Compared to previous studies, the proportion of patients showing visuospatial impairment was relatively low. This may be ascribed to the stringent criteria of *z*‐score < − 2.0 we adopted. A similar trend was replicated with lenient criteria of *z*‐score < − 1.0. Our study explores a critical link between VOR and visuospatial dysfunction that could affect daily activities and quality of life in patients with PD.

### 4.2 The Major Role of the VOR: Maintenance of Balance

The major role of the VOR is to maintain balance; in general, the risk of falls increases when a patient has vestibular deficits (Wuehr et al. 2022). However, it remains under debate whether the VOR is affected during the disease course of PD: Deficient VOR can be found in patients with PD during the disease course, approximately in 30% of patients (Hawkins et al. 2022; Hong et al. 2024). Alternatively, no changes were noted compared with age‐ and sex‐matched healthy controls (Hawkins et al. 2022).

Furthermore, in contrast to conventional wisdom, the association between the vestibular system and balance is at odds in patients with PD. The afferent pathways, such as the VOR and vestibulospinal reflex, have little significance for balance in the presence of a deficient efferent pathway (the extrapyramidal system) (Hawkins et al. 2022; Hong, Baik, et al. 2024). Rather, the severity of the efferent pathway may determine the risk of falls in patients with PD. Reportedly, vestibular rehabilitation (do Amaral et al. 2024) and galvanic stimulation (Mahmud et al. 2022) may have the potential to ameliorate the postural imbalance in PD, and concrete evidence is required to elucidate the role of the VOR or VSR in maintaining balance in PD.

### 4.3 Role of the VOR in Visuospatial Cognition

Spatial orientation and navigation are complex processes that integrate sensory information from multiple modalities to update orientation in space relative to known external space locations (Glasauer et al. 2002). Both egocentric and allocentric processes update the internal representations of position and orientation in space. This internal representation of external coordinates depends on linear and rotational vestibular inputs from the otolith and canal signals, respectively (Muir et al. 2009; Yoder and Taube 2009). Thus, age‐related decline in the semicircular canal and utricular function leads to deficient spatial orientation (Anson et al. 2021).

Likewise, in healthy participants, lower VOR gain for the HC was associated with poorer performance on RCFT‐copying in our patients (Oh et al. 2024). Generally, RCFT‐copying reflects visuoconstructional ability, whereas RCFT‐delayed recall largely depends on nonverbal memory. Our findings suggest that vestibular information may play a crucial role in visuospatial perception and construction, in addition to its main role in controlling balance in PD (Angelaki et al. 2009). This association is valid irrespective of the motor symptoms of PD or cognitive reserve in our patients. Similarly, deterioration of the VOR leads to deranged self‐motion perception and spatial disorientation (Anson et al. 2021). Patients with unilateral or bilateral vestibulopathies also show impairments in visuospatial cognitive tests (Bosmans et al. 2022; Oh et al. 2024; Oh et al. 2023; Zhang et al. 2022); hence, our findings suggest that the VOR affects visuospatial cognition in patients with PD.

The RCFT‐delayed recall was not associated with the VOR gain for any canal in our patients. Given that verbal and visuospatial memory dysfunction can occur since the early stage of PD (Aarsland et al. 2017), the contribution of the VOR to visuospatial memory may not be as robust as the role for visuospatial perception. Instead, our findings suggest that visuospatial memory is largely determined by the MMSE as a representative of the general cognitive reserve in PD.

### 4.4 Speculation of the Neural Substrates and Routes Responsible for Spatial Perception: Vestibular Network

Our study cannot answer which neural structure or at what level the interaction between spatial perception and the VOR occurs. Brandt et al. demonstrated that visuospatial learning and memory depend largely on vestibular information (Brandt et al. 2005). Patients showed normal memory performance on standardized neuropsychological tests following bilateral vestibular neurectomy. However, patients exhibited deficient visual learning and memory in the virtual Morris water task, correlating with the degree of hippocampal atrophy on MRIs (Brandt et al. 2005). The task was conducted using a laptop monitor devoid of vestibular stimuli. Notably, patient performance was surprisingly impaired compared with that of healthy participants. This result suggests that hippocampal processing of spatial memory relies primarily on vestibular inputs while stationary (Brandt et al. 2005). Numerous animal studies also support this clinical result, wherein temporary inactivation of the vestibular system disrupts location‐specific firing in hippocampal place cells in rats (Stackman et al. 2002). In addition, other brain areas related to memory, such as the thalamic head‐direction and entorhinal grid cells, are known to have vestibular connections (Muir et al. 2009; Yoder and Taube 2009). Vestibular‐thalamic projections to the vestibular and entorhinal cortices provide a basis for self‐motion perception and spatial orientation, respectively (Cullen and Chacron 2023). Thus, vestibular signals contribute to the hippocampal spatial representation.

The deficit may not be localized to a certain neural structure, such as the hippocampus, but is ascribed to the dysfunction of the vestibular network. Functional alterations have been noted in the basal ganglia and limbic structures, showing visuospatial deficits in patients with PD (Grahn et al. 2009). Patients with visuospatial deficits show reduced activation of the right insula and hippocampus as well as the left putamen and bilateral caudate nuclei (Caproni et al. 2014). Likewise, patients with visuospatial dysfunction show reduced gray matter volume in the fusiform, right parahippocampal, and middle occipital gyri (Pereira et al. 2009). Vestibular information may facilitate visuospatial perception at any level in the vestibular cortex, thereby enabling the interpretation and perception of internal and external representations of the self and environment.

### 4.5 Relationship Between the VOR and Spatial Disorientation in PD and its Relevance for Clinicians

Our findings suggest that vestibular function may particularly affect the neural representation of spatial information and that spatial disorientation is correlated with the degree of bilateral vestibulopathy (Schöberl et al. 2021). Thus, the navigation strategy differs in patients with vestibulopathy (Gammeri et al. 2022). Chronic bilateral vestibular loss decreases reliance on allocentric reference frames associated with hippocampal and entorhinal activations, whereas unilateral vestibular loss decreases the likelihood of using egocentric reference frames associated with the fronto‐temporo‐parietal network (Gammeri et al. 2022).

Interestingly, RCFT‐copying was associated with the VOR gain for the HC but not the VOR gain for the vertical canals. This may be ascribed to differences in visual exploration during the stationary and locomotive states, wherein patients usually utilize horizontal visual exploration in a quiet stationary or upright stance, directing the VOR and saccadic eye movements preferably along the horizontal plane (Dieterich and Brandt 2024). By contrast, during locomotion, visual exploration occurs primarily along the vertical plane (Hollands et al. 1995). As the RCFT was conducted while our patients were seated, the VOR for the HC may have shown relevance but not that for the vertical canals. Visual exploration tasks during locomotion or dual tasks, such as cognitive assessment while balancing on a force platform covered with a foam pad, may yield different results (Danneels et al. 2023). Furthermore, the influence of the AC may be masked by overestimation caused by axial rigidity in our study design (Woo et al. 2024).

### 4.6 Other Factors That Could Affect the Visuospatial Function and Cognition in PD

The cognitive function can be affected by various factors in PD. Patients with bulbar dysfunction, hallucination, and autonomic dysfunction have a greater chance of developing cognitive impairment (Uc et al. 2009). Depression can greatly impair short‐term memory in PD (Norman et al. 2002). The social functioning, referring to an individual's ability to live independently and interact with society, can also have a close link with cognitive dysfunction in PD (Chen et al. 2022; Su et al. 2020; Yager and Ehmann 2006).

Notably, the visuospatial dysfunction is associated with the disease burden of PD, having a close relationship with disease duration, certain phenotypes (e.g., postural instability and gait difficulty subtype), or motor severity (Lally et al. 2020; Levin et al. 1991; Sahakian et al. 1988). In this context, previous studies have primarily focused on finding connections with motor symptoms. Our study further demonstrates that the VOR function, an often‐overlooked ability, can be associated with visuospatial perception and learning in PD. Incorporating other possible factors may inspire future studies to further explore the relationship between VOR function and visuospatial ability, ultimately contributing to strategies that enhance the real‐world quality of life for patients with PD.

### 4.7 Caveats and Limitations of Our Study and Suggestions for Future Studies

A major limitation of our study is that it is a cross‐sectional study, and the association between the VOR and visuospatial ability was not observed in the age‐ and sex‐matched controls. Our findings cannot answer whether this association is valid in healthy older individuals. A longitudinal follow‐up study can provide a deeper understanding of the association. Second, our results also did not establish a direct causal relationship between spatial orientation and the VOR function. Vestibular dysfunction may contribute to functional impairment and the generation of spatial disorientation, thereby hampering outdoor activities (Whitney et al. 2004). In contrast, patients with spatial disorientation generally have decreased mobility and social functioning, exhibiting little chance of vestibular adaptation and/or multisensory integration (Black and Pesznecker 2003; Kim et al. 2025; Lee et al. 2024; Yager and Ehmann 2006). Third, this study was conducted in a patient cohort consisting of an Asian population, which limits the generalizability of our findings. Future research should incorporate additional physiological factors, such as genetic influences (e.g., race‐specific genes), as well as other biological, psychological, and social factors, to enhance the comprehensiveness of these findings (Chang et al. 2024; Fang et al. 2019; Morley et al. 2012).

Despite these limitations, our findings can influence therapeutic interventions or diagnostic approaches for assessing the cognitive function in PD. For example, wearable sensors and virtual reality devices are available for clinical and home use to accurately measure the motor and non‐motor symptoms (e.g., social functioning and spatial orientation), as well as vestibular rehabilitation (Battista and Romaniello 2024; Hong 2024; Monje et al. 2019; Yu et al. 2022). Utilizing these advanced technologies to assess and rehabilitate VOR function can potentially improve the visuospatial dysfunction in PD. Moreover, the relationship between VOR and other cognitive subdomains, beyond visuospatial learning and memory, deserves further investigation. The findings of this study can assist in diagnosing cognitive disorders within the PD population and may pave the way for developing new diagnostic tools to enhance clinical effectiveness.

We explored a critical connection between VOR and visuospatial dysfunction in patients with PD, highlighting a significant non‐motor symptom that affects daily activities and quality of life. Our findings suggest the VOR function may be associated with visuospatial perception and learning in PD. This implicates the development of more targeted therapeutic interventions and offers insights into the broader implications of PD on sensory‐motor integration and cognitive function.

## Author Contributions

**Yukang Kim**: writing–original draft, writing–review and editing, methodology. **Tonghoon Woo**: writing–review and editing, software, data curation. **Seoui Kwag**: data curation, methodology. **Hyunsoh Park**: data curation, methodology. **Hanseob Kim**: data curation, formal analysis, validation. **Kyoungwon Baik**: data curation, formal analysis, visualization, writing–review and editing. **Sun‐Uk Lee**: writing–review and editing, writing–original draft, conceptualization, methodology, data curation, supervision, funding acquisition, project administration, formal analysis, visualization. **Euyhyun Park**: writing–review and editing, formal analysis. **Chan‐Nyoung Lee**: writing–review and editing, data curation, supervision. **Gerard J. Kim**: supervision, data curation, writing–review and editing. **Ji‐Soo Kim**: writing–review and editing, data curation, methodology, visualization.

## Ethics Statement

This study followed the tenets of the Declaration of Helsinki and was performed according to the guidelines of the Institutional Review Board of Korea University Anam Hospital (2023AN0442).

## Conflicts of Interest

The authors declare no conflicts of interest.

## Peer Review

The peer review history for this article is available at https://publons.com/publon/10.1002/brb3.70453

## Supporting information



Supplementary Table 1. Sensitivity analysis of prediction of abnormal RCFT‐copying defined as z‐score < 1.0
Supplementary Table 2. Sensitivity analysis of predicting abnormal RCFT‐copying using VOR gain of each canal as nominal variables

## Data Availability

The data that support the findings of this study are available from the corresponding author upon reasonable request.
