# Supplementary material for: The Vestibulo‐Ocular Reflex is Associated With Visuospatial Dysfunction in Patients With Parkinson's Disease
Source: Brain Behav. 2025 Apr 1;15(4):e70453. doi: 10.1002/brb3.70453 (PMC12498219; doi:10.1002/brb3.70453)
Supplement: Supplementary file 1 — Supplementary Table 1. Sensitivity analysis of prediction of abnormal RCFT‐copying defined as z‐score < 1.0 Supplementary Table 2. Sensitivity analysis of predicting abnormal RCFT‐copying using VOR gain of each canal as nominal variables [file BRB3-15-e70453-s001.docx]

**Supplementary Table 1. Sensitivity analysis of prediction of abnormal RCFT-copying defined as z-score <**$\boldsymbol{-}$**1.0**

| Variables | Univariate analysis | | Multivariable analysis | |
| --- | --- | --- | --- | --- |
|  | **OR (95% CI)** | ***p*-value** | **OR (95% CI)** | ***p*-value** |
| Age, years | 1.05 (1.01–1.10) | 0.025 |  |  |
| Sex, female | 0.65 (0.30–1.44) | 0.287 |  |  |
| Disease duration, months^a^ | 1.00 (0.98–1.02) | 0.931 | 0.96 (0.92–1.00) | 0.041 |
| MDS-UPDRS-III | 1.03 (1.00–1.06) | 0.065 |  |  |
| MDS-UPDRS-III-kinetic tremor^b^ | 1.40 (0.85–2.33) | 0.191 |  |  |
| Orthostatic hypotension | 1.30 (0.59–2.90) | 0.519 |  |  |
| Years of schooling, years | 0.86 (0.78–0.94) | 0.002 | 0.83 (0.72 – 0.96) | 0.014 |
| MMSE | 0.76 (0.66–0.88) | <0.001 |  |  |
| SCOPA-PC | 1.10 (0.85–1.43) | 0.472 |  |  |
| Depression | 0.83 (0.31–2.22) | 0.704 |  |  |
| Anxiety | 0.67 (0.18–2.44) | 0.540 |  |  |
| Cadence, steps/min | 1.01 (0.98–1.05) | 0.581 |  |  |
| VOR gain, HC | 0.03 (0.001–0.74) | 0.032 | 0.001 (0.001–0.10) | 0.004 |
| VOR gain, AC | 0.42 (0.05–3.82) | 0.441 |  |  |
| VOR gain, PC | 0.21 (0.02–1.98) | 0.174 |  |  |

^a^Disease duration refers to the period from onset of motor symptoms to presentation.

^b^in dominant hand

**Supplementary Table 2. Sensitivity analysis of predicting abnormal RCFT-copying using VOR gain of each canal as nominal variables**

| Variables | Univariate analysis | | Multivariable analysis | |
| --- | --- | --- | --- | --- |
|  | **OR (95% CI)** | ***p*-value** | **OR (95% CI)** | ***p*-value** |
| Age, years | 1.05 (0.98–1.13) | 0.195 |  |  |
| Sex, female | 0.21 (0.04–1.01) | 0.051 | 0.14 (0.01–1.30) | 0.083 |
| Disease duration, months^a^ | 1.01(0.99–1.04) | 0.363 |  |  |
| MDS-UPDRS-III | 1.04 (0.99–1.08) | 0.150 |  |  |
| MDS-UPDRS-III-kinetic tremor^b^ | 1.51 (0.71–3.22) | 0.280 |  |  |
| Orthostatic hypotension | 0.97 (0.27–3.48) | 0.965 |  |  |
| Years of schooling, years | 0.93 (0.81–1.07) | 0.293 |  |  |
| MMSE | 0.75 (0.62–0.89) | 0.001 |  |  |
| SCOPA-PC | 1.18 (0.83–1.66) | 0.360 |  |  |
| Depression | 0.35 (0.04–2.85) | 0.328 |  |  |
| Anxiety | 0.68 (0.08–5.62) | 0.718 |  |  |
| Cadence, steps/min | 1.05 (0.99–1.12) | 0.098 |  |  |
| Decreased VOR gain, HC | 4.14 (1.10–15.62) | 0.036 | 10.79 (1.94–60.11) | 0.007 |
| Decreased VOR gain, AC | 3.24 (0.61–17.26) | 0.169 |  |  |
| Decreased VOR gain, PC | 2.61 (0.50–13.59) | 0.256 |  |  |

^a^Disease duration refers to the period from onset of motor symptoms to presentation.

^b^in dominant hand
